# TAVI na América Latina – Chegaremos Lá!

**DOI:** 10.36660/abc.20220325

**Published:** 2022-06-06

**Authors:** Silvio Gioppato, Rodrigo Modolo

**Affiliations:** 1 Universidade de Campinas Faculdade de Ciências Médicas Divisão de Cardiologia Campinas SP Brasil Departamento de Medicina Interna – Divisão de Cardiologia, Faculdade de Ciências Médicas. Universidade de Campinas – UNICAMP, Campinas, SP – Brasil; 2 Boston Scientific Corporation Maple Grove EUA Boston Scientific Corporation, Maple Grove – EUA

**Keywords:** Estenose da Valva Aórtica, Valva Aórtica/anormalidades, Substituição da Valva Aórtica, Transcateter/métodos, Intervenção Coronária Percutânea/métodos

A exemplo da intervenção coronária percutânea, o Implante Transcateter de Válvula Aórtica (TAVI) caminha a passos largos na direção de superar a abordagem cirúrgica e se tornar o procedimento predominante no tratamento da estenose aórtica. Desde o primeiro implante percutâneo feito pela equipe do Dr. Alain Cribier, que completa 2 décadas este ano, o TAVI vem demonstrando, estudo após estudo, robustas evidências da sua eficácia e segurança.^1^ Em cada etapa desta jornada, os desafios foram sendo sucessivamente superados tanto pelo aperfeiçoamento dos dispositivos como pelos aprendizados adquiridos pelos operadores, o que permitiu avançar de forma consistente do cenário do risco cirúrgico proibitivo até o de baixo risco em pouco mais de 15 anos.^2-7^ E a evolução é ininterrupta^8-10^ – estudos em andamento investigam a expansão do TAVI para populações de pacientes jovens, valva aórtica bicúspide, assintomáticos e mesmo na insuficiência aórtica pura.

Hoje a pergunta de 1 milhão de dólares é sobre a durabilidade dos dispositivos que, em parte, começa a ser respondida. Em 2019, Thyregod et al.,^11^ publicaram o resultado de 5 anos do estudo NOTION (The Nordic Aortic Valve Intervention Trial) mostrando não haver diferenças tanto no objetivo primário composto de morte por qualquer causa, AVC ou infarto (TAVR 38% x SAVR 36%; p=0,86) como nos eventos individuais. Mais recentemente, no congresso do American College of Cardiology (ACC 2022), Michael Reardon apresentou os resultados de 5 anos combinando os estudos CoreValve US Pivotal e SURTAVI mostrando que em pacientes de risco intermediário ou alto a taxa de deterioração estrutural da válvula foi significativamente menor no grupo TAVI comparado ao grupo cirúrgico (2,57% x 4,38%; p=0,0095). Mas esses dados ainda são insuficientes para responder se o TAVI será o Padrão Ouro no tratamento das doenças da valva aórtica, independentemente de etiologia, idade ou tipo de disfunção.

Aspecto também importante é o financiamento da tecnologia. Sempre que surge um avanço tecnológico com segurança e eficácia comprovadas por estudos clínicos, ocorre um choque entre evidência e custo da tecnologia, gerando um debate que acaba consumindo tempo entre a consolidação das evidências e a incorporação da tecnologia aos sistemas de saúde do mundo todo. Contudo, nos países em desenvolvimento, esse embate é ainda mais alongado, criando um paradoxo no qual a tecnologia está presente na prática médica, mas inacessível à maior parte da população por anos. Tal descompasso estabelece um distanciamento entre as realidades dos países desenvolvidos e em desenvolvimento no que tange ao volume de procedimentos, número de centros capacitados, expertise dos operadores e a disponibilidade de diferentes dispositivos.

Com olhar voltado para o tema, nesta edição dos Arquivos Brasileiros de Cardiologia, Bernardini et al.,^12^ buscaram como objetivo primário comparar a prática da TAVI entre centros latino-americanos e do resto do mundo a partir de dados da pesquisa WRITTEN 2015 que abarcou 250 centros no mundo todo, sendo 29 na América Latina (AL), aqui representados como LATAM 15, e 221 nos demais países e continentes (WORLD 15). A pesquisa consistiu num questionário composto por 59 perguntas abrangendo diferentes domínios do TAVI que foi enviado para diversos centros no mundo todo, cuja decisão de participação foi espontânea e voluntária. Como objetivo secundário os autores buscaram avaliar também a evolução na prática da TAVI na AL após 5 anos através de uma nova rodada do questionário no continente em 2020 (LATAM 20).

Os resultados não surpreendem quando comparados ao resto do mundo, sendo observado que na AL a experiência cumulativa e o volume anual de procedimentos foram muito menores (mediana 34 vs. 200; p<0,001) refletindo o hiato existente entre países desenvolvidos e aqueles em desenvolvimento. Entretanto, há um lado positivo observado neste estudo que mostra uma aproximação das práticas dos centros LATAM 20 com os centros WORLD 15. Vale destacar o crescimento, mesmo sem atingir diferença estatística, de quase duas vezes no volume de procedimentos dos centros LATAM 20 comparado aos LATAM 15 (mediana 62 vs. 34; p=0,08), o aumento significativo na proporção de pacientes de risco cirúrgico intermediário e baixo (15,2% vs. 21,2% e 2,2% vs. 6,4%, respectivamente, p=0,04) e o aumento significativo no número de centros realizando procedimentos transfemorais com sedação consciente/anestesia local (LATAM 15 4% x LATAM 20 11%; p<0,001). A aproximação das práticas também aparece nas condutas peri e pós-procedimento e de seguimento, as quais nos centros LATAM 20 estão em compasso com aquelas observadas nos centros WORLD 15.

O fato de os achados estarem lastreados em informações retrospectivas, fornecidas por meio de questionários facultativos e não compulsórios, fragilizam a extrapolação das interpretações. Mas não enfraquecem a visão de que o descompasso entre a AL e o resto do mundo existe e precisa ser considerado pelas autoridades de saúde pública desses países. Os autores também reforçam a importância do trabalho de educação continuada desenvolvido pelas sociedades médicas em parceria com a indústria para o crescimento e aperfeiçoamento consistente da técnica no nosso meio.
