# Supplementary material for: TAVI na América Latina – Chegaremos Lá!
Source: Arq Bras Cardiol. 2022 Jun 6;118(6):1097–8. [Article in Portuguese] doi: 10.36660/abc.20220325 (PMC9345157; doi:10.36660/abc.20220325)
Supplement: Translation

# Translation: Transcatheter Aortic Valve Implant in Latin America – We will Get There!

**Authors:** Silvio Gioppato, Rodrigo Modolo

**Affiliations:** 1 Campinas SP Brazil Departamento de Medicina Interna – Divisão de Cardiologia, Faculdade de Ciências Médicas. Universidade de Campinas – UNICAMP, Campinas, SP – Brazil; 2 Maple Grove USA Boston Scientific Corporation, Maple Grove – USA

**Keywords:** Aortic Valve Stenosis, Aortic Valve/abnormalities, Transcatheter Aortic Valve Replacement/methods, Percutaneous Coronary Intervention/methods

As with percutaneous coronary intervention, the Transcatheter Aortic Valve Implantation (TAVI) is making great strides toward overcoming the surgical approach and becoming the predominant procedure in treating aortic stenosis. Since the first percutaneous implantation made by Dr. Alain Cribier, who turns 2 decades old this year, TAVI has demonstrated, study after study, robust evidence of its efficacy and safety.^1^ At each stage of this journey, the challenges were successively overcome both by the improvement of the devices and the skills acquired by the operators, which allowed us to move consistently from the prohibitive surgical risk scenario to the low-risk scenario in just over 15 years.^2-7^ Furthermore, evolution is unfolding^8-10^ – ongoing studies investigate the expansion of TAVI for young patient populations, bicuspid aortic valve, asymptomatic and even pure aortic regurgitation.

Today the million-dollar question is about the durability of devices that, in part, starts to be answered. In 2019, Thyregod et al.^11^ published the 5-year result of the NOTION study (The Nordic Aortic Valve Intervention Trial), showing no differences in either the primary composite endpoint of death from any cause, stroke or heart attack (TAVR 38% vs. SAVR 36%; p=0.86) as in individual events. More recently, at the American College of Cardiology Congress (ACC 2022), Michael Reardon presented the 5-year results combining the CoreValve US Pivotal and SURTAVI studies showing that in intermediate- or high-risk patients, the rate of valve structural deterioration was significantly lower in the TAVI group compared to the surgical group (2.57% x 4.38%; p=0.0095). Nevertheless, these data are still insufficient to answer whether TAVI will be the Gold Standard for treating aortic valve diseases, regardless of etiology, age or type of dysfunction.

Another important aspect is the financing of technology. Whenever a technological advance appears with safety and efficacy proven by clinical studies, there is a clash between evidence and the cost of technology, generating a debate that ends up consuming time between the consolidation of evidence and the incorporation of technology into health systems around the world. However, this struggle is even longer in developing countries, creating a paradox in which technology is present in medical practice but inaccessible to most of the population for years. Such a mismatch establishes a gap between the realities of developed and developing countries in terms of procedure volume, number of trained centers, operators’ expertise, and the availability of different devices.

With an eye on the subject, in this edition of *Arquivos Brasileiros de Cardiologia*, Bernardini et al.^12^ sought as a primary objective to compare the TAVI practice between Latin American centers and the rest of the world, based on data from the WRITTEN 2015 survey that covered 250 centers worldwide, being 29 in Latin America, represented here as LATAM 15, and 221 in other countries and continents (WORLD 15). The research consisted of a questionnaire composed of 59 questions covering different TAVI domains sent to several centers worldwide whose decision to participate was spontaneous and voluntary. As a secondary objective, the authors also sought to assess the evolution of TAVI practice in Latin America (LA) after 5 years through a new round of the questionnaire on the continent in 2020 (LATAM 20).

The results are not surprising when compared to the rest of the world, noting that in LA, the cumulative experience and the annual volume of procedures were much lower (median 34 vs. 200; p<0.001), reflecting the gap between developed and developing countries. However, there is a positive side observed in this study, which shows an approximation of the practices of the LATAM 20 centers with the WORLD 15 centers. It is worth mentioning the nearly 2-fold increase in procedure volume comparing the 5-year period between 2015 and 2020 in LA – event though not statistically significant – (median 62 vs 34 procedures; p=0.08); the significant increase in the proportion of patients with intermediate and low surgical risk (15.2% vs 21.2% and 2.2% vs 6.4%, respectively for LATAM 15 and 20, p=0,04) and the significant increase in the number of centers performing transfemoral procedures with conscious sedation/local anesthesia (LATAM 15 4% x LATAM 20 11%; P<0.001). The approximation of practices also appears in the peri- and post-procedure and follow-up procedures, which in the LATAM 20 centers are in step with those observed in the WORLD 15 centers.

The fact that the findings are backed by retrospective information, provided through optional and non-compulsory questionnaires, weakens the extrapolation of interpretations. However, they do not weaken the view that the mismatch between LA and the rest of the world exists and needs to be addressed by public health authorities in these countries. The authors also reinforce the importance of continuing education work developed by medical societies in partnership with industry for the consistent growth and improvement of the technique in our LA countries
